# Defense mechanisms promoting tolerance to aggressive *Phytophthora* species in hybrid poplar

**DOI:** 10.3389/fpls.2022.1018272

**Published:** 2022-10-13

**Authors:** Martin Cerny, Miroslav Berka, Miloň Dvořák, Ivan Milenković, Iñigo Saiz-Fernández, Břetislav Brzobohatý, Jaroslav Ďurkovič

**Affiliations:** ^1^ Department of Molecular Biology and Radiobiology, Faculty of AgriSciences, Phytophthora Research Centre, Mendel University in Brno, Brno, Czechia; ^2^ Department of Forest Protection and Wildlife Management, Faculty of Forestry and Wood Technology, Phytophthora Research Centre, Mendel University in Brno, Brno, Czechia; ^3^ Department of Forestry, University of Belgrade-Faculty of Forestry, Belgrade, Serbia; ^4^ Department of Phytology, Technical University in Zvolen, Zvolen, Slovakia

**Keywords:** metabolome, lipidome, *Phytophthora plurivora*, *Phytophthora cactorum*, *Populus*, biotic interaction, proteome

## Abstract

Poplars are among the fastest-growing trees and significant resources in agriculture and forestry. However, rapid growth requires a large water consumption, and irrigation water provides a natural means for pathogen spread. That includes members of *Phytophthora* spp. that have proven to be a global enemy to forests. With the known adaptability to new hosts, it is only a matter of time for more aggressive *Phytophthora* species to become a threat to poplar forests and plantations. Here, the effects of artificial inoculation with two different representatives of aggressive species (*P. cactorum* and *P. plurivora*) were analyzed in the proteome of the *Phytophthora*-tolerant hybrid poplar clone T-14 [*Populus tremula* L. 70 × (*Populus × canescens* (Ait.) Sm. 23)]. Wood microcore samples were collected at the active necrosis borders to provide insight into the molecular processes underlying the observed tolerance to *Phytophthora*. The analysis revealed the impact of *Phytophthora* on poplar primary and secondary metabolism, including carbohydrate-active enzymes, amino acid biosynthesis, phenolic metabolism, and lipid metabolism, all of which were confirmed by consecutive metabolome and lipidome profiling. Modulations of enzymes indicating systemic response were confirmed by the analysis of leaf proteome, and sampling of wood microcores in distal locations revealed proteins with abundance correlating with proximity to the infection, including germin-like proteins, components of proteosynthesis, glutamate carboxypeptidase, and an enzyme that likely promotes anthocyanin stability. Finally, the identified *Phytophthora-*responsive proteins were compared to those previously found in trees with compromised defense against *Phytophthora*, namely, *Quercus* spp. and *Castanea sativa*. That provided a subset of candidate markers of *Phytophthora* tolerance, including certain ribosomal proteins, auxin metabolism enzymes, dioxygenases, polyphenol oxidases, trehalose-phosphate synthase, mannose-1-phosphate guanylyltransferase, and rhamnose biosynthetic enzymes. In summary, this analysis provided the first insight into the molecular mechanisms of hybrid poplar defense against *Phytophthora* and identified prospective targets for improving *Phytophthora* tolerance in trees.

## Introduction

The genus *Populus* is composed of more than two dozen different species separated into at least four distinct clades and native to most of the Northern Hemisphere (Wang et al., 2020). Poplars are among the fastest-growing trees in temperate regions and have become significant resources in agriculture and forestry (He et al., 2018). The natural poplar forests cover more than 50 million ha, predominantly in Russia, Canada, the United States, and China (FAO, 2016). The area of poplar plantations reached 31 million ha in 2016, and with rotation lengths of 5 to 15 years, poplars, and especially hybrid poplars, are one of the top three most planted trees worldwide (Nicolescu et al., 2020; Rebola-Lichtenberg et al., 2021). Besides providing an important source of biofuel and biomass (Monson et al., 2020), poplar planting has more beneficial roles in the environment. For example, poplars are used for the planting of field shelterbelts that can regulate and reduce surface runoff. Due to the expansive root system, poplar trees act as green filters and have been successfully used for phytoremediation (Castro-Rodríguez et al., 2016; Ancona et al., 2019; Barra Caracciolo et al., 2020).

Most poplars are sensitive to stress, including salt stress (Sixto, 2005; Zhang et al., 2019), cold (Yang et al., 2019), and heat (Tozzi et al., 2013). However, among the abiotic stressors, water availability is the most limiting one. The rapid growth of poplar is highly dependent on water, and drought results in rapid decline and mortality (Xi et al., 2021). Besides abiotic stressors, poplars are susceptible to diseases. In line with the trade-off theory, rapid growth is correlated with lower defense mechanisms and a relatively short lifespan, at least compared with long-lived trees (Piovesan and Biondi, 2021). Poplars are hosts to a plethora of microbes and opportunistic pathogens (Biselli et al., 2022), and poplar’s susceptibility to phytopathogens is the main obstacle to its mass cultivation and exploitation (Isebrands and Richardson, 2014; Li et al., 2019; Ji et al., 2020; Kwaśna et al., 2021).

*Phytophthora* diseases are yet to cause significant losses in the poplar population. However, these microbes have been causing increasing problems for many tree species (Jung et al., 2018). *Phytophthora* can adapt rapidly to new hosts, and the worldwide trade is increasing the risk of introducing new and more aggressive *Phytophthora* populations. Taken together with a high demand for irrigation that provides natural means for *Phytophthora* spreading, poplar plantations are facing a serious threat. Indeed, recent reports have shown that *Phytophthora* species are already present in the rhizosphere under poplar trees, including aggressive species such as *P. cactorum* and *P. plurivora* (Keča et al., 2016; Milenković et al., 2018). Following an artificial inoculation, both these pathogens induced a canker formation in the hybrid poplar clone T14 (Ďurkovič et al., 2021). Interestingly, the necrotic tissue was eventually sequestered, and *Phytophthora* hyphae were prevented from penetrating deeper into the healthy living tissues. Compared to other poplar trees grown in the area, the wood of the hybrid poplar clone T-14 contained a lower amount of lignin and a higher amount of cellulose (Ďurkovič et al., 2017; 2020). However, the mechanisms of *Phytophthora* tolerance have not been reported.

Here, we analyzed hybrid poplar wood microcores collected thirteen months post-inoculation at the border of the active necrosis site and identified proteins involved in molecular mechanisms responsible for preventing the *Phytophthora* invasion. We then observed the spatial distribution of these proteins by analyzing microcores collected in zones up to 20 cm from the active necrosis border. Next, we searched for systemic response in the leaf proteome. Finally, we performed wood metabolome and lipidome profiling to validate our proteomics results. The presented analyses provided novel insights into the plant-pathogen interaction, and the comparison with previously published data pinpointed putative targets for improving tree resistance against *Phytophthora*. It should be noted that both *Phytophthora* spp. had been previously found in the region and were considered wide spread (Pánek et al., 2016; Tkaczyk et al., 2020). The introduction of alien invasive pathogens did not occur, and thus the experiment did not pose a threat to the local environment.

## Materials and methods

### Plant material, oomycete isolates and sampling

**Figure 1:**
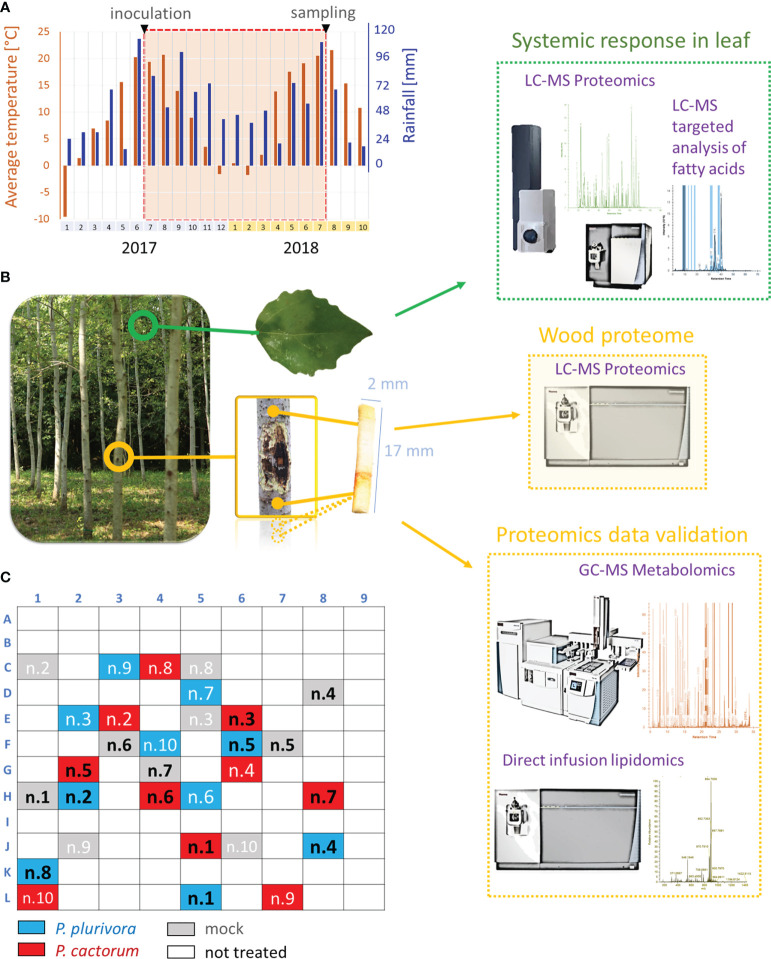
Overview of the experiments. **(A)** Rainfalls and average monthly temperature of the field study site during the experiment. **(B)** Omics experiments. Wood omics analyses (yellow) were complemented by analysis of leaf proteome and fatty acid pool (green). GC-MS metabolomics profiling and lipidome analysis were used for proteome data validations. **(C)** Diagram representing the location of trees used in the experiment. Samples in black were collected for omics analyses.

In June 2017, ten-year-old micropropagated plants of the hybrid poplar clone T-14 [*Populus tremula* L. 70 × (*Populus* × *canescens* (Ait.) Sm. 23)] were artificially inoculated using the standardized underbark inoculation test at breast height, i.e., 130 cm above the tree base (Ďurkovič et al., 2021). The *P. cactorum* isolate SFB057 (GenBank accession number JX276094) and the *P. plurivora* isolate SFB182 (GenBank accession number KF234740) used for inoculation were both isolated from the planted poplar stand in Serbia (44°39’52” N; 19°59’43” E; 80 m a.s.l.) and represent aggressive *Phytophthora* species of similar pathogenicity routinely used in bioassays (e.g., Taylor and Grünwald, 2021). Isolates were grown for 3-5 days on V8 juice agar (V8A) media [100 ml l^-1^ of V8 juice (Pfanner, GmbH), 3 g l^–1^ CaCO_3_, 20 g l^–1^ of agar (Sigma-Aldrich), 900 ml l^–1^ of distilled water; Milenković et al., 2018], and square plugs 1.5 × 1.5 cm in size were taken from the edges of actively growing colonies and used as inocula. Thirty hybrid poplar trees were randomly selected within the experimental field and inoculated with *Phytophthora* or mock. In brief, after the surface sterilization with ethanol and flame, bark pieces 1.5 × 1.5 cm in size were carefully cut and removed using the sterilized scalpel, and the topsides of inocula were placed on the exposed wood. The inoculation site was covered with sterile cotton moistened in distilled water and sealed with Parafilm M (Bemis) and aluminum foil. The mock-treated control plants received the same treatments using agar plugs of the same size. All plants used for the study grew in the experimental field plot at Zvolen, Slovakia (**Figures 1A–C**; 48°35′N; 19°08′E; 297 m a.s.l.). The distribution of inoculated trees and the climatic conditions of the site are indicated in **Figure 1**. No unprecedented temperature or drought extremes were observed within the period of the experiment. It should be noted that the surrounding area was regularly monitored for symptoms of *Phytophthora* infection, and no bark cankers or defoliations were found (**Figure 1**).

Wood microcores were sampled thirteen months post-inoculation, i.e., at the time when the bark cankers were already established on tree trunks and the infected trees responded visibly to the disease by the lateral callus formation. Two microcores per each of five biological replicates (1.7 cm in length, 0.2 cm in diameter) were sampled from around the bark cankers, approximately 1.5 cm from the active necrosis borders at both the upstream and downstream regions of the inoculation site. For the same trees, additional samples were collected at 0, 3, 10, and 20 cm from the active necrosis borders. In addition, leaves from the bottom branches were sampled at the height of approximately 350 cm. The collected samples were flash-frozen in liquid nitrogen, ground to a fine powder using a Retsch Mill MM400, and stored at –80°C until analysis. *Phytophthora* re-isolations were performed by plating the necrotic bark fragments onto selective V8A-PARPNH media (Milenković et al., 2018), taken from the transition zones between necrotic and apparently healthy tissues at both upper and lower necrosis margins.

### Scanning electron microscopy

Wood samples were collected at the interface between the active necrosis region and healthy bark tissue, approximately 3 cm from the inoculation site. Radial surfaces of hybrid poplar wood sections (0.4 × 0.4 cm) were mounted on specimen stubs, sputter-coated with gold in the Sputter Coater K650X (Quorum Technologies, Ashford, UK) in an argon atmosphere, and examined by high-vacuum scanning electron microscopy (SEM) using a JEOL JSM-6390 LV instrument (JEOL, Tokyo, Japan) operating at 20 kV. Three samples per treatment were analyzed.

### Proteomic analysis

Approximately 100 mg of homogenized tissue were extracted for omics analyses as described previously (Berka et al., 2020b; Saiz-Fernández et al., 2020), and portions of samples corresponding to 5 µg of peptide were analyzed by nanoflow reverse-phase liquid chromatography-mass spectrometry using a 15 cm C18 Zorbax column (Agilent), a Dionex Ultimate 3000 RSLC nano-UPLC system and the Orbitrap Fusion Lumos Tribrid Mass Spectrometer (Thermo Fisher; one technical replicate per sample) or a qTOF maXis Impact mass spectrometer (Bruker; two technical replicates per sample). The measured spectra were recalibrated and searched against the *P. trichocarpa* 4.1 (Tuskan et al., 2006), *P. tremula* × *P. alba hybrid* (sPta717 v2), *P. cactorum*, *P. plurivora* (Vetukuri et al., 2018; Yang et al., 2018), and common contaminants databases, using Proteome Discoverer 2.5 (Thermo Fisher). The quantitative differences were determined by Minora, employing precursor ion quantification followed by normalization (total area) and calculation of relative peptide/protein abundances. For qTOF data, the spectral counting method was employed (Dufková et al., 2019), followed by normalization (total PSMs count) and ANOVA with Tukey’s HSD (MetaboAnalyst; Pang et al., 2021). Only proteins with at least two unique peptides and minimum of 10 peptide spectral matches were considered for the analysis. Proteomaps were generated using the online tool at http://bionic-vis.biologie.uni-greifswald.de (Liebermeister et al., 2014). The representative mass spectrometry proteomics data have been deposited to the ProteomeXchange Consortium *via* the PRIDE partner repository (Perez-Riverol et al., 2022) with the dataset identifiers PXD035956 and PXD035965.

### Metabolomic and lipidomic analyses

Samples from collected wood microcores (one technical replicate per tree listed in **Figure 1C**) were extracted and fractionated with tert-butyl methyl ether:methanol mixture, aliquots were derivatized and measured using a Q Exactive GC Orbitrap GC-tandem mass spectrometer and Trace 1300 Gas chromatograph (Thermo Fisher) as described in Berka et al. (2020a) and Saiz-Fernández et al. (2020). Data were analyzed by Compound Discoverer 3.2 (Thermo Fisher) and searched against NIST2014, GC-Orbitrap Metabolomics library, and in-house library. Only metabolites fulfilling identification criteria (score ≥ 75 and ΔRI < 5%) were included in the final list. The lipid fraction was analyzed as previously described (Dufková et al., 2022). In brief, samples were dried by vacuum centrifugation, redissolved in 200 μL isopropanol/methanol/tert-butyl methyl ether 4/2/1 (v/v/v) with 20 mM ammonium formate, and analyzed by direct infusion employing Triversa Nanomate (Advion Biosciences) nanoelectrospray source and the Orbitrap Fusion Lumos Mass Spectrometer. The acquired profile spectra were analyzed using FreeStyle 1.7 and LipidSearch 4.2 (Thermo Fisher). In parallel, leaf fatty acid content in aliquots of collected leaf tissue samples (one technical replicate per tree, **Figure 1C**) was evaluated as described previously (Cerna et al., 2017), using TSQ Quantiva (Thermo Fisher) and Skyline 3.1 (Pino et al., 2020).

### Annotation of proteins and cross-species comparisons

Protein functional annotations were obtained by searching protein sequences against *Arabidopsis thaliana *proteome using STRING 11.0. The annotation for proteins that did not match any Arabidopsis orthologs were found using UniProt database BLAST (https://www.uniprot.org/blast). The cross-species comparisons were based on data found in two large proteomics datasets describing proteome changes near the inoculation point (Saiz-Fernández et al., 2020; Saiz-Fernández et al., 2022): (i) *P. cinnamomi* isolate TJ 349, root inoculation of micropropagated *Quercus variabilis* and *Quercus suber* plants, root tissue collected at five time points following the infection progress; (ii) *P. cinnamomi* isolate TJ 349, stem inoculation of two-year-old *Castanea sativa* plants, stem sections collected right at the end of the necrotic tissue and approximately 20 cm upstream of that area). The matching proteins were selected based on three factors: Arabidopsis gene identifiers for matched orthologs, enzymatic activity for proteins with assigned Enzyme Commission (EC) number, and protein family identity based on conserved features in the protein sequence (UniProt). The *Phytophthora* response was classified as similar, contrasting, and unique for proteins showing a similar trend, contrasting response, and those found only in poplar proteome, respectively. For proteins matching multiple orthologs, the similar response indicates the identification of at least one ortholog with a similar response.

### Statistical analysis

The reported statistical tests were generated and implemented as follows using default and recommended settings unless otherwise indicated. The reliability of protein and metabolite identifications were assessed in Proteome Discoverer 2.5 (Thermo Fisher Scientific) and Compound Discoverer 3.2 (Thermo Fisher Scientific). Student’s t-test was calculated using MS Excel. For ANOVA with Tukey’s HSD and Kruskal-Wallis tests, Instant Clue (Nolte et al., 2018), the Real Statistics Resource Pack software for MS Excel (Release 6.8; Copyright 2013 – 2020; Charles Zaiontz; www.real-statistics.com), and MetaboAnalyst 5.0 (Pang et al., 2021) were employed. The GO enrichment, estimations of pathway impact, and interactions were determined using ShinyGO 0.76 (Ge et al., 2020) and STRING 11.0 (Szklarczyk et al., 2019). Intraomics interactions were analyzed by OmicsAnalyst (Zhou et al., 2021), using Spearman’s rank correlation. PCAs were performed in ProteomeDiscoverer and CompoundDiscoverer, using normalized abundances and default scaling. OPLS and VIP were performed in SIMCA 14.1 (Sartorius). Significant differences refer to p<0.05, unless otherwise stated.

## Results

### Both pathogens were detected in the active necrosis site by electron microscopy and re-isolations

**Figure 2:**
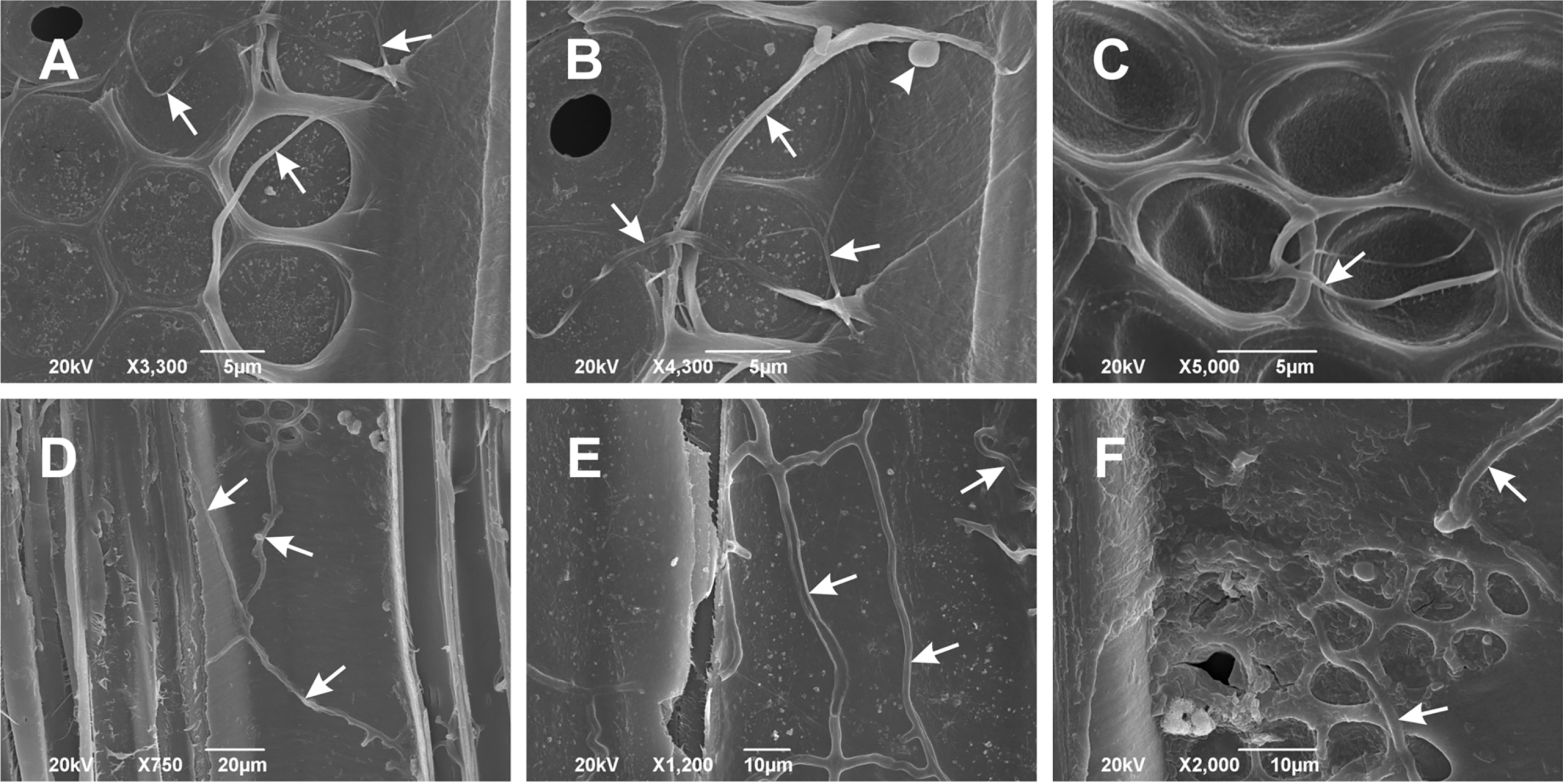
Scanning electron microscopy images of *Phytophthora cactorum* **(A–C)** and *Phytophthora plurivora* **(D–F)** hyphae in the outermost annual growth ring of inoculated hybrid poplar plants. Images show radial vessel sections with *Phytophthora* hyphae (arrows) spreading on the radial vessel wall surface. Arrowhead in **(B)** points to the globose oogonium in *P*. *cactorum*. Radial sections, scale bars: **(A–C)** = 5 μm, **(D)** = 20 μm, **(E, F)** = 10 μm. Representative images out of three replicates collected thirteen months after the inoculation.

The effects of inoculation were monitored at regular intervals of three to four weeks, and the pathogenicity results, assessed 51 and 154 days after the inoculation, were reported elsewhere (Ďurkovič et al., 2021). For this study that was undertaken thirteen months after the inoculation, the outermost annual growth ring was analyzed, and the presence of *Phytophthora* hyphae was confirmed by both SEM (**Figures 2A–F**) and re-isolations. At this stage, the infected trees began to close the gaps of bark cankers, indicating that the cambial division actively promoted a defensive lateral callus formation.

### Wood proteome analysis of the samples collected at the border of the active necrosis showed similarity in response to *P. plurivora* and *P. cactorum*

Five biological replicates were sampled (**Figure 1C**) by drilling the wood tissue in the proximity of active necrosis. Total protein extracts were analyzed, and measured spectra were searched against the protein databases of *P. trichocarpa* 4.1 and *P. tremula* × *P. alba* hybrid (sPta717 v2). The final set contained 4765 protein families represented by 29812 peptides. The searches against the *P. cactorum* and *P. plurivora* databases found only peptides matching three putative *P. plurivora* proteins. However, none of these proteins were exclusively found in inoculated samples, indicating a false positive assignment (Berka et al., 2020a).

**Figure 3:**
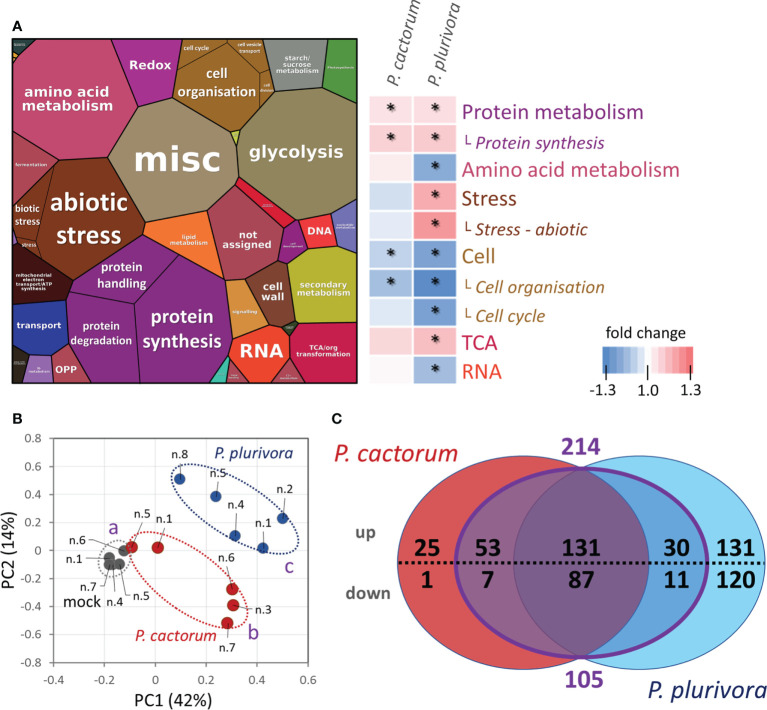
Response of hybrid poplar proteome sampled at the site of actively growing necrosis. **(A)** The composition of wood proteome visualized by ProteoMap and corresponding heatmap summarizing significant differences in response to *Phytophthora*. The size of a protein in the ProteoMap corresponds to the estimated mean content, protein annotations and functional analysis are based on Arabidopsis orthologs, and color-coding in the heatmap category names corresponds to the ProteoMap visualization. Asterisks indicate statistically significant differences compared to mock-treated plants (p < 0.05).. **(B)** Differences in proteome composition visualized by Principal Component Analysis. Statistically significant differences (Kruskal-Wallis, p<0.05) between *Phytophthora* (red, blue) and mock-inoculated trees (gray) are indicated. Based on proteome abundances of all differentially abundant proteins. **(C)** The comparison of differentially abundant proteins (absolute fold change FC > 1.4; p < 0.05) in response to *P. cactorum* and *P. plurivora* visualized in a Venn diagram. The purple area represents proteins that did not show statistically significant differences between *P. cactorum* and *P. plurivora* responses. Results are based on five biological replicates, numbers correspond to **Figure 1C.** For details, see **Supplementary Table S1**.

Next, proteomes of infected and mock-treated samples were compared. The quantitative differences based on abundances of 3123 proteins identified with at least two unique peptides and representing more than 95% of the estimated protein content were analyzed by ProteoMaps using annotations of the corresponding Arabidopsis orthologs (**Figure 3A**). The consecutive pair-wise comparison highlighted a statistically significant (p<0.05) increase in protein metabolism and a decrease in proteins involved in cell organization. *Phytophthora plurivora* had a much higher impact on the plant proteome, resulting in a significant accumulation of stress-related proteins and proteins of TCA metabolism, and a decrease in enzymes associated with amino acid metabolism and RNA metabolism. There was also a decrease in fatty acid metabolism by 9% on average, but the statistical significance was lower (p<0.1).

The detailed analysis of individual proteins found 596 differentially abundant proteins (ANOVA, Tukey’s HSD; **Table S1**) corresponding to approximately 10% of the estimated protein content in the mock-inoculated samples. The analysis showed a disparity between samples infected with *P. cactorum* and *P. plurivora* (**Figures 3B, C**). In total, 304 and 510 putative *Phytophthora*-responsive proteins were found in *P. cactorum* and *P. plurivora* samples, respectively. Of these, a similar response was found for 218 proteins. An additional subset of 101 *Phytophthora*-responsive proteins did not show statistically significant differences between *P. cactorum* and *P. plurivora* datasets, being statistically different from the mock-treated controls in only one of the treatments. Interestingly, none of these 319 proteins manifested a contrasting response to different species of *Phytophthora*, indicating a high degree of similarity in response to the oomycota pathogens.

### *Phytophthora-*responsive proteins in wood indicated modulation in primary and secondary metabolism

**Figure 4:**
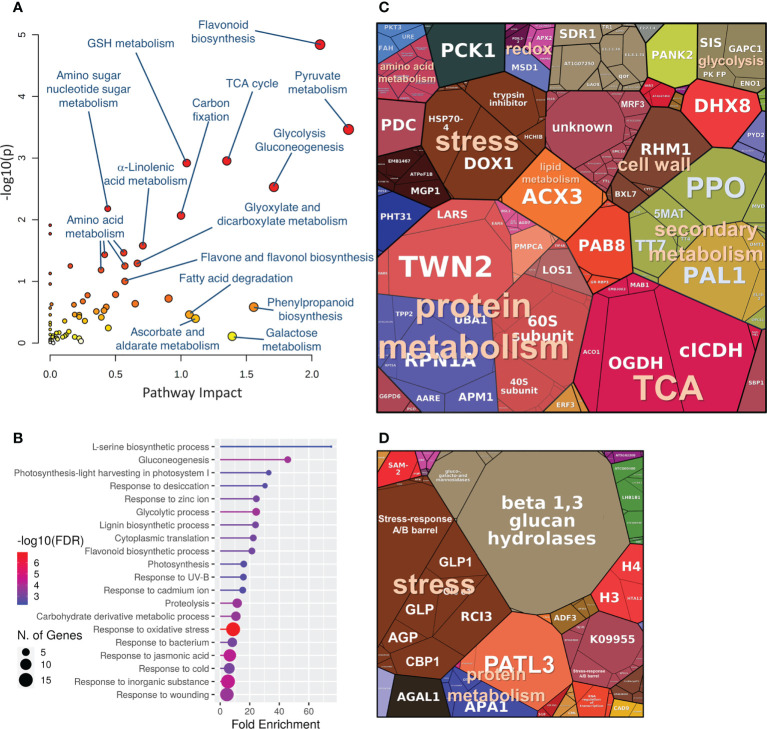
The expected role of differentially abundant proteins found in hybrid poplar wood proteome in the proximity of *Phytophthora*-induced necrosis. **(A, B)** Functional enrichment of corresponding Arabidopsis orthologs visualized by MetaboAnalyst 5.0 and ShinyGO 0.76). Proteomap visualization of significantly accumulated **(C)** and depleted **(D)** *Phytophthora-*responsive proteins. The size of a protein in the ProteoMap corresponds to the estimated mean content in the mock-treated plants. The scale of ProteoMaps is identical. For details, see **Supplementary Table S1**.

Hybrid poplar proteins that showed a similar response to *P. cactorum* and *P. plurivora* were mapped to 185 and 86 Arabidopsis orthologs for accumulated and depleted proteins, respectively. The analysis of the gene ontology enrichment and metabolic pathway impact highlighted a statistically significant impact on secondary metabolism, including flavonoid biosynthesis, phenylpropanoid biosynthesis, and lignin biosynthetic process. Significant enrichment was also found for pyruvate metabolism and TCA cycle, carbohydrate metabolism (carbohydrate-active enzymes, CAZymes), response to ROS, amino acid metabolism, and fatty acid metabolism (**Figures 4A, B**). The consecutive ProteoMap visualization (**Figure 4C**) showed that the most abundant proteins that accumulated in response to necrosis belonged to protein metabolism (tRNA ligases, ribosomal proteins, elongation factors, components of protein targeting and ubiquitination pathway), energy metabolism (citric acid metabolism enzymes, glycolytic enzymes, enzymes involved in lipid degradation, components of mitochondrial respiratory chain), secondary metabolism (enzymes of isoprenoid, phenylpropanoids, and flavonoids biosynthesis, polyphenol oxidases), stress response (HSP70, oxylipin metabolism enzyme, PR proteins, ROS metabolism enzymes), amino acid metabolism, and cell wall metabolism. The annotation of putative Arabidopsis orthologs of proteins that were significantly less abundant in proximity to necrosis (**Figure 4D**) indicated a decrease in photosynthesis, modulation of histones, and a decrease in cell wall loosening (ortholog of alpha-galactosidase GAL1). Interestingly, there was a decrease in proteins associated with defense, including proteases (aspartate protease, two cysteine proteases, and serin protease), an ortholog of peroxidase RCI3, several germin-like proteins, and a putative glucan endo-1,3-beta-glucosidase.

There were only 26 proteins that seemed to be specific for *P. cactorum* response (**Table S1**), including a member of the cytochrome P450 family and several proteins with a putative role in cell wall protection and maintenance (polygalacturonase inhibitor, inhibits the function of cell wall pectin degrading enzymes; an ortholog of Arabidopsis subtilase AT5G67360, triggers the accumulation and/or activation of cell wall modifying enzymes; ortholog of RHD3, required for cell wall biosynthesis and actin organization). In contrast, *P. plurivora-*specific response was found for 251 proteins. Based on the annotations of Arabidopsis orthologs, 58 of these proteins represented different isoforms of enzymes that were found to be affected in response to both *Phytophthora* pathogens. These included, for example, three out of five *Phytophthora*-responsive polyphenol oxidases (3/5), cinnamyl alcohol dehydrogenases (2/7), peroxidases (7/11), and aspartic proteases (4/6). For proteins that are not classified as enzymes, the comparison of protein families showed additional 23 proteins that likely represented a similar response. The most numerous were orthologs of actin-depolymerizing factor (3/4), HSP70 family proteins (5/6), and lipid-transfer proteins (4/5). The subset of 170 P*. plurivora* responsive-proteins that did not seem to match any *P. cactorum* response was enriched in sHSP family proteins (18), serine carboxypeptidases (7), nucleoredoxin (4), and proteins involved in intracellular vesicle trafficking and protein transport (rab family proteins, HSP90-7 endoplasmin homolog, SNARE proteins, vesicle-associated protein VAMP714). Metabolic pathway analysis showed a significant impact (p<0.05) on purine/pyrimidine metabolism, lipid metabolism, and starch and sucrose metabolism.

### Spatial distribution of *Phytophthora-*responsive proteins

**Figure 5:**
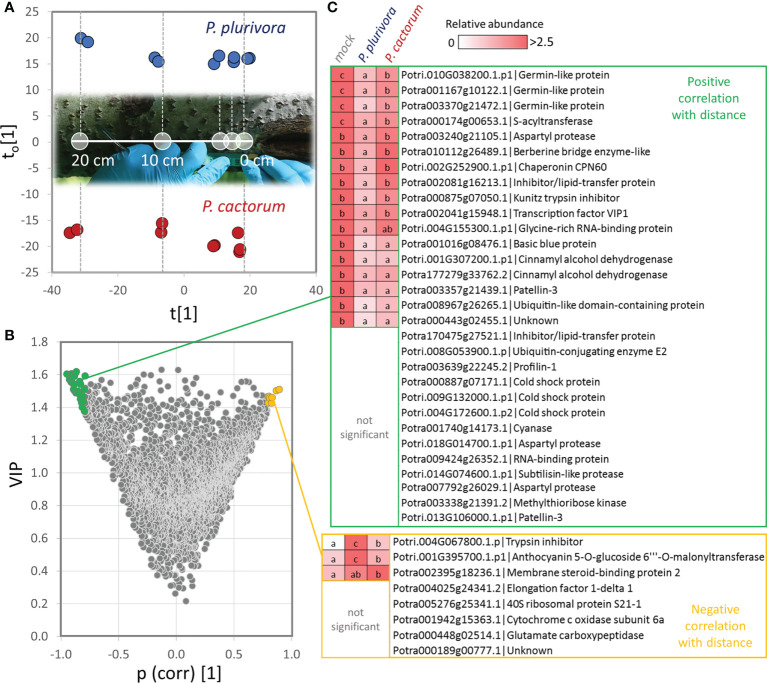
Identification of proteins that showed correlation with distance to the necrosis site. **(A)** Orthogonal partial least squares discriminant analysis followed by **(B)** VIP (variables of importance in projection). **(C)** Identified proteins with the most significant positive (pcorr[1]>0.8, VIP>1.4; green) and negative (pcorr[1]<-0.8, VIP>1.4; yellow) correlation of abundance with the distance to the necrosis site. Heat-map visualization and letters represent significant differences (p <0.05, ANOVA, Tukey’s HSD) at the distance of 1.5 cm. For details, see **Supplementary Tables S1** and **S2**.

The wood proteome analysis revealed nearly 600 putative *Phytophthora-*responsive proteins. To provide evidence for their role in response to infection, wood tissue of selected representative trees was sampled at 0, 1.5, 3, 10, and 20 cm distance relative to the site of the active necrosis border. The obtained quantitative data for 3059 proteins were assessed *via* an orthogonal partial least squares (OPLS) regression analysis (**Figures 5A–C**; **Table S2**), and the correlation between distance from the necrosis boundaries and protein abundances was evaluated. In total, 181 proteins showed the expected accumulation patterns, increasing (pcorr[1]>0.5) and decreasing (pcorr[1]<-0.5) abundance in the proximity to the necrosis, for accumulated and depleted proteins, respectively. That represents 30% of all identified differentially abundant proteins. Indeed, most proteins of the set did not meet the 0.5 thresholds. However, only three proteins were found to accumulate in the pattern that was opposite to the expected one. The set of proteins that showed a very high correlation (pcorr[1]>0.8) and were accumulated close to the infection zone included putative malonyl transferase that acylates anthocyanins and promotes anthocyanin stability (Suzuki et al., 2002), components of proteosynthesis, an ortholog of glutamate carboxypeptidase that has a role in signaling and defense response (e.g., Lee et al., 2016), protease inhibitor, an unknown membrane protein binding steroid compounds, and a subunit of cytochrome c oxidase complex (**Figure 5C**). Very high negative correlations (pcorr[1]<-0.8) were found for 30 proteins, including multiple germin-like protein isoforms, cinnamyl alcohol dehydrogenases, proteases, and protein inhibitors (**Figure 5C**).

### Effects of *Phytophthora* infection on wood metabolome

**Figure 6:**
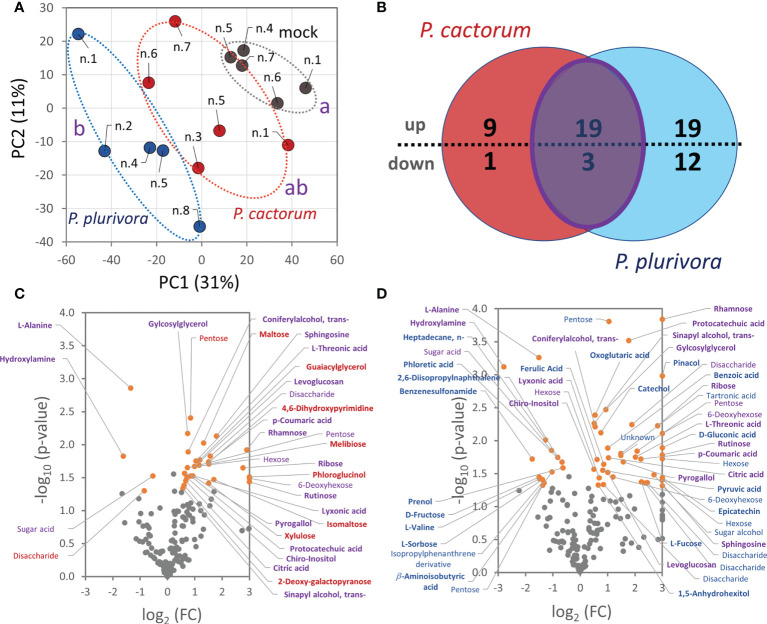
*Phytophthora* infection altered hybrid poplar metabolome in the areas surrounding the necrotic tissue. **(A)** Differences in wood metabolome composition visualized by Principal Component Analysis. Statistically significant differences (Kruskal-Wallis, p<0.05) between *Phytophthora* (red, blue) and mock-inoculated trees (gray) are indicated. Based on normalized abundances of all resolved metabolite features (25000 TIC threshold). **(B)** The comparison of differentially abundant metabolites (absolute fold change FC > 1.4; p < 0.05) in response to *P. cactorum* and *P. plurivora* visualized in a Venn diagram and corresponding volcano plots **(C, D)**. Similarities (purple) and *Phytophthora-*specific responses (red, blue) are highlighted. Results are based on five biological replicates, numbers corresponds to **Figure 1C**. For details, see **Supplementary Table S3**.

Polar metabolites were profiled by GC-MS untargeted analysis and more than 2700 features were resolved. The clustering of metabolome profiles showed a similarity to the separation of proteomes, with the *P. plurivora* infected samples being significantly separated in the first component of PCA from the control ones (**Figure 6A**). In total, 170 compounds passed the detection criteria filter and 63 showed statistically significant differences in response to *Phytophthora*. An accumulation was found for 19 compounds, including 5 phenolics, 11 carbohydrates, chiro-inositol, sphingosine, and citric acid (**Figure 6B**). Hydroxylamine, alanine, and an unidentified sugar acid were significantly depleted. A notable increase was also found for sucrose. The 1.2-fold change did not meet the set general threshold, but sucrose is the main carbohydrate that represents more than 40% of the metabolome of the analyzed wood, so the observed accumulation seemed biologically relevant. *Phytophthora cactorum*- and *P. plurivora*-specific response was found for 10 and 31 metabolites, respectively (**Figures 6C, D**; **Table S3**). The metabolites that specifically accumulated only in response to *P. cactorum* were carbohydrates and two phenolic compounds (phloroglucinol and a metabolite annotated as guaiacylglycerol). An unknown disaccharide was less abundant in these samples. Besides phenolics and carbohydrates, *P. plurivora* response induced accumulation of additional citric acid cycle metabolites (pyruvate, oxoglutaric acid) and flavonoids (catechol and epicatechin). Metabolites of interest included, among others, phloretic acid, a product of flavonoid degradation. It seems that its decrease could correlate with flavonoid accumulation.

### Systemic response in leaves

**Figure 7:**
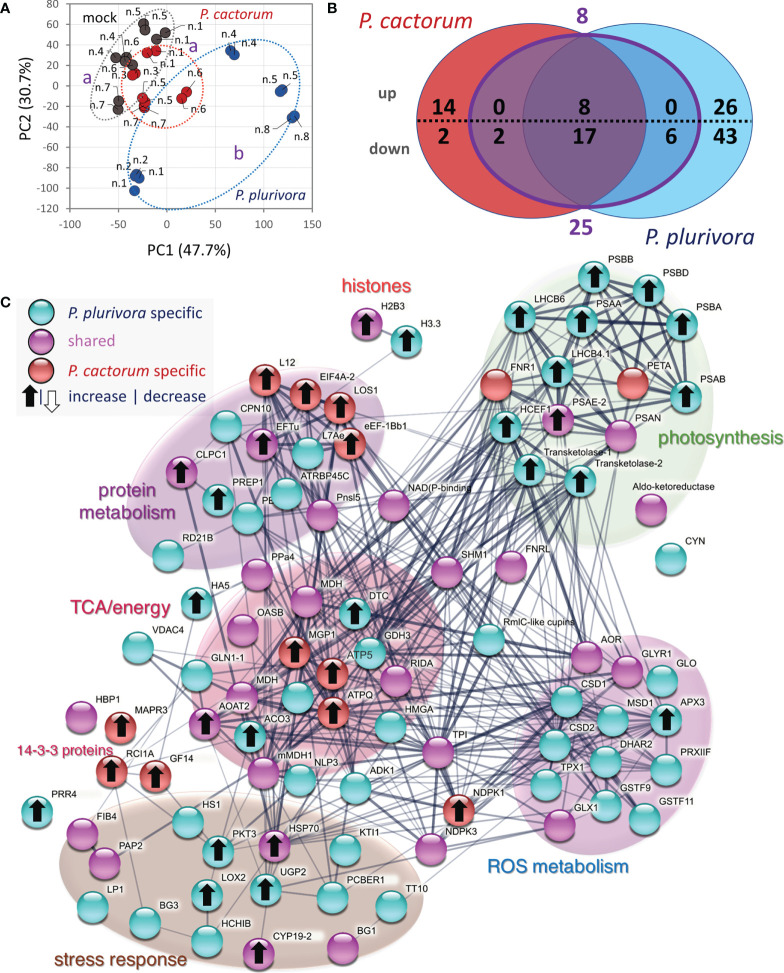
Systemic response in leaf proteome. **(A)** Differences in proteome composition visualized by Principal Component Analysis. Statistically significant differences (Kruskal-Wallis, p<0.05) between leaf proteome of *P. plurivora* (blue), *P. cactorum* (red), and mock-inoculated trees (gray) are indicated. Based on proteome abundances of all differentially abundant proteins. **(B)** The comparison of differentially abundant proteins (absolute fold change FC > 1.4; p < 0.05) in response to *P. cactorum* and *P. plurivora* visualized in a Venn diagram. Results are based on five biological replicates analyzed in two technical replicates, numbers correspond to **Figure 1C**. **(C)** Interactions and functional clusters of *Phytophthora*-responsive proteins highlighted by String. For details, see **Supplementary Table S4**.

The employed analysis of leaf proteome was less sensitive, allowing identification and confident quantitation of only 1414 and 708 most abundant proteins, respectively. Interestingly, the response found in the wood microcore proteome was reflected in the composition of the leaf proteome (**Figures 7A–C**), indicating a certain level of systemic response and alteration of metabolism induced by *Phytophthora*. The comparison with mock-inoculated trees revealed 118 differentially abundant proteins. A similar response to *P. cactorum* and *P. plurivora* was found for 33 proteins, but only 25 of these passed a 1.4-fold change threshold in response to both pathogens. The comparison with the wood proteome dataset showed 17 shared differentially abundant proteins, but only orthologs of a chloroplastic multi-pass membrane protein RER4, elongation factor LOS1, ATP synthase subunit MGP1, membrane steroid-binding protein MAPR3, and lipid-transfer protein LP1 had a similar response to that found in the wood proteome (**Table S5**). A similar response was also found for members of the HSP70 family similar to Arabidopsis HSP70-4. HSP70 proteins have an important role in plant immunity (Berka et al., 2022) but the available data indicate that the accumulated leaf isoforms were not identical to those found in the wood tissue. The *Phytophthora* infection had a negative impact on the abundance of a subset of stress-responsive proteins in leaves, including orthologs of beta-1,3-glucanase (BG11; a negative role in callose deposition; Zavaliev et al., 2013) and plastid-lipid associated protein FIB4 (role in plastoglobule development and stress resistance, Singh et al., 2010). A decrease in the abundance was also found for proteins of the citric acid cycle, amino acid metabolism, energy metabolism, and ROS metabolism, which was a contrasting response to that found in wood microcores. The effect of *P. plurivora* was stronger, but it seemed to be targeting similar metabolic pathways. For example, both *Phytophthora* species elicited an increase in an ortholog of histone protein H2B3, but only *P. plurivora* induced accumulation of histone H3.3. Similarly, not one but two isoforms of beta-1,3-glucanase were decreased in response to *P. plurivora*. A contrasting effect seemed to be found in proteins of photosynthetic apparatus, indicating promoted photosynthetic activity in leaves of *P. plurivora* infected trees. There was also an apparent increase in the jasmonic acid metabolism, indicated by an accumulation of 3-ketoacyl-CoA thiolase (PKT3) and lipoxygenase (LOX2). *Phytophthora cactorum-*specific response of interest was found for two 14-3-3 proteins (key members of signal transduction cascades, e.g., Hloušková et al., 2019).

### Lipid composition reflected the proximity of *Phytophthora* infection and evidenced systemic response in leaves

**Figure 8:**
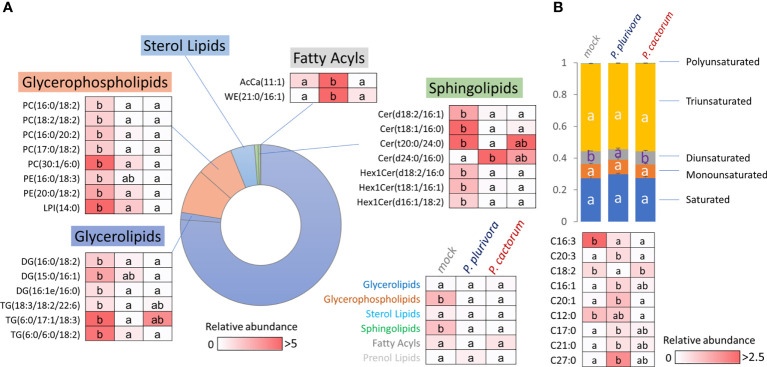
Lipid profile of wood in the proximity of necrosis **(A)** and free fatty acid pool in leaves **(B)**. The impact of *Phytophthora* on lipidome composition and identified differentially abundant lipids (Kruskal-Wallis, p<0.05). AcCa, acyl carnitine; Cer, cermaide; DG, diglyceride; Hex1Cer, Simple Glc series; LPI, lysophosphatidylglycerol; PC, phosphatidylcholine; PE, phosphatidylethanolamine; TG, triglyceride; WE, wax ester. For details, see **Supplementary Tables S5**, **S6**.

Proteome analysis revealed a putative effect of *Phytophthora* infection on lipidome composition. In order to validate that, the lipidome profile was analyzed by the direct infusion method, and more than 400 lipid species were detected. Fragmentation spectra provided confident identification of 275 lipid compounds, and the consecutive statistical analysis found 26 differentially abundant lipids (p<0.05, 1.5-fold change threshold; **Figure 8A**, **Supplementary Table S5**). The most abundant compounds identified in the wood microcore extracts were representatives of triglycerides, phosphatidylcholines, diglycerides, and phosphatidylethanolamines, covering more than 90% of the estimated lipid abundance. *Phytophthora* had a statistically significant impact on the total pool of glycerophospholipids and sphingolipids (significantly depleted in response to infection). Six identified *Phytophthora*-responsive glycerolipids were also significantly less abundant, but these represented only a minor fraction of storage lipids (6% of the mock-treated samples; **Figure 8A**). Acyl carnitine (11:1) and wax ester (21:0/16:1) showed a statistically significant response only in the presence of *P. plurivora*, but the remaining 21 compounds showed a similar response to both pathogens.

Next, the wood microcore lipidome profiling was supplemented with the analysis of the free fatty acid pools of the leaves and quantitative data was obtained for 27 major fatty acids. The comparison with mock-treated controls showed that there was a significant decrease in diunsaturated fatty acids in *P. plurivora*-infected trees, and the corresponding ratio between diunsaturated:saturated fatty acids dropped by 30%. Detailed analysis of quantified fatty acids showed nine putative *Phytophthora*-responsive fatty acids (**Figure 8B**), revealing an accumulation of three unsaturated fatty acids, one monounsaturated C16:1 fatty acid, and a decrease in the C16:3 fatty acid pool. None of the differentially abundant fatty acids was specific for *P. cactorum*, and only three were found to be specific for *P. plurivora* response, including linoleic acid (C18:2, a significant decrease).

### Integration of omics data highlighted role of phenolics and germins in response to *Phytophthora*

**Figure 9:**
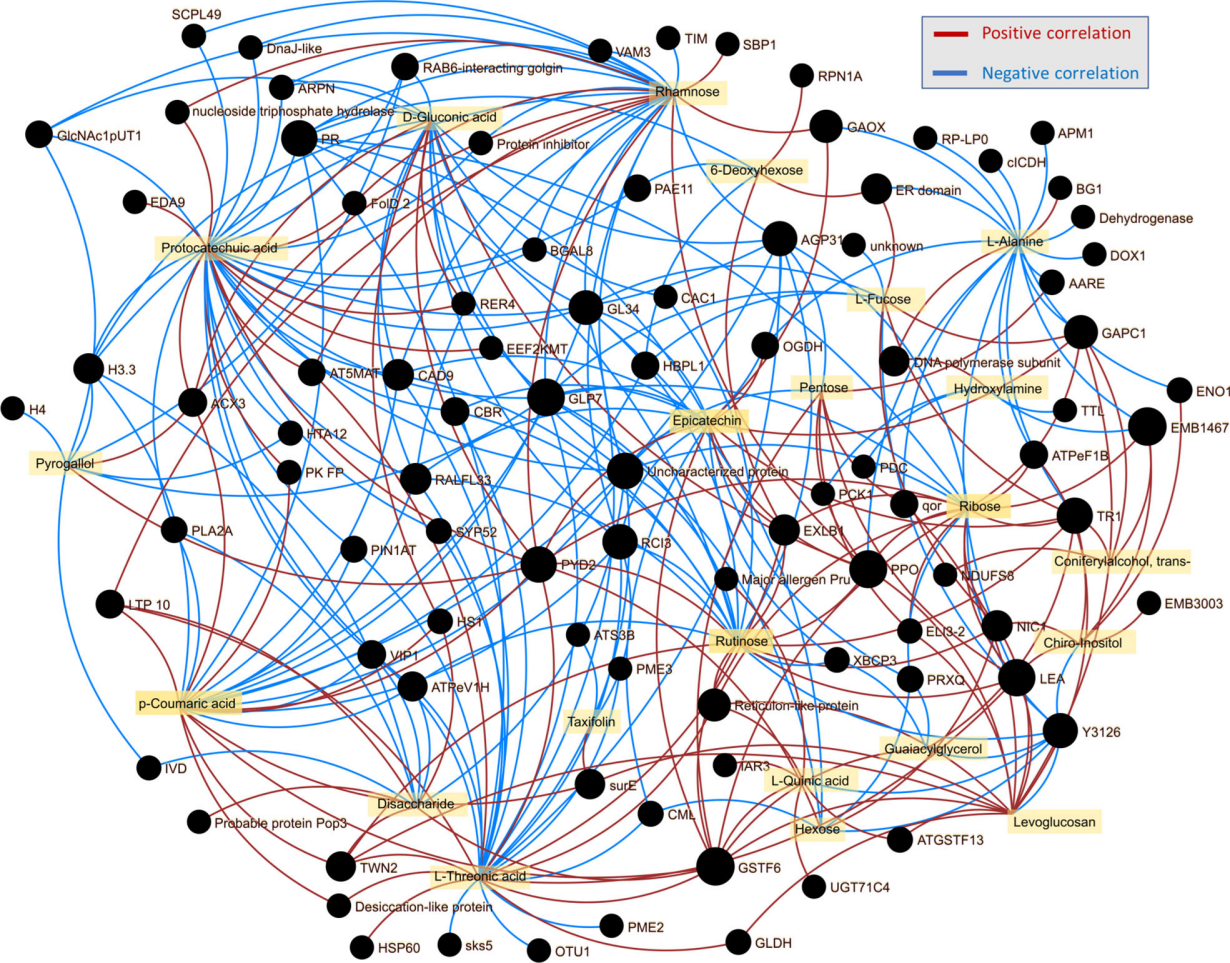
Integration of omics data indicated the role of phenolic compounds in shaping wood proteome response to *Phytophthora*. Omics data interaction was assessed by OmicsAnalyst, employing Spearman’s rank correlation threshold 0.85 for intraomics interactions.

The integration of omics data is known to reveal connections and provide novel insights into molecular mechanisms (Pascual et al., 2017; Valledor et al., 2021). Here, the analysis of wood proteome and metabolome revealed several clusters of proteins and metabolites that showed similar accumulation patterns in response to *Phytophthora*. The list contained 26 metabolites and 118 proteins that passed criteria for intra-omics interactions with absolute Spearman’s rank correlations r_s_>0.85 (**Figure 9**, **Supplementary Table S7**). The highest number of intra-omics interaction was found for protocatechuic acid (32 proteins), threonic acid (29), rhamnose (27), and coumaric acid (23). The impact of phenolic compound metabolism was also reflected in epicatechin (20) and rutinose (22), a disaccharide that is present in some flavonoid glycosides. Four germins (Potri.010G038200.1.p1, Potra001167g10122.1, Potra003370g21472.1, and Potri.012G111500.1.p1), glutathione S-transferase GSTF6 (Potri.002G207093.1.p), dihydropyrimidinase (Potri.009G067700.1.p1), a peroxidase (Potri.017G038100.1.p1), pathogenesis related protein (Potri.009G028800.1.p1), and a polyphenol oxidase (Potra152996g26723.1) were the most frequent interactors, which is well-in-line with the presumed role in stress responses. The integration of lipidomics data showed only two small clusters indicating putative correlations of 15 lipids with 25 proteins (**Supplementary Figure S1**). The highlighted proteins did not appear to be related to lipid metabolism or signaling. The only correlation that is less likely to have occurred simply by chance is that of phospholipase 2A (Potri.019G015100.1.p1, a decrease in abundance in response to *P. plurivora*). Phospholipase is reportedly regulated by sphingolipids (Nakamura et al., 2010), and its abundance was correlated with two ceramides [Cer(t20:0/24:0), a positive correlation; Cer(d24:0/16:0), a negative correlation].

### The comparison of hybrid poplar *Phytophthora-*responsive proteins with proteins found in response to *P. cinnamomi*

**Figure 10:**
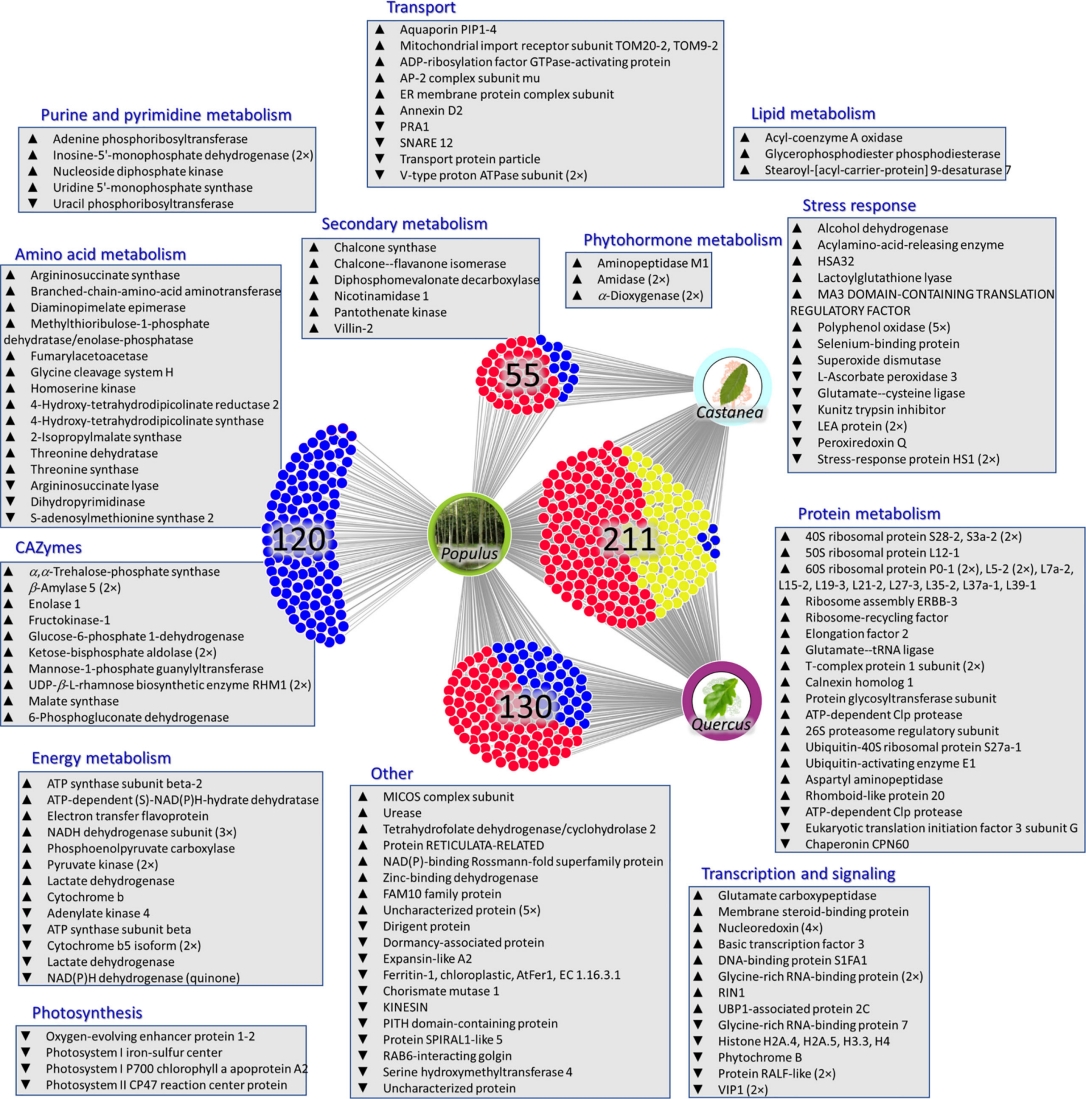
Putative mechanisms promoting tolerance against *Phytophthora.* The comparison of hybrid poplar *Phytophthora-*responsive proteins with proteins found in response to *P. cinnamomi* in *Quercus* spp. (*P. cinnamomi* isolate TJ 349, root inoculation of micropropagated *Q. variabilis* and *Q. suber* plants, root tissue collected at five time points following the infection progress; Saiz-Fernández et al., 2022) and *Castanea sativa* (*P. cinnamomi* isolate TJ349, stem inoculation of two-year-old *Castanea sativa* plants, stem sections collected right at the end of the necrotic tissue and approximately 20 cm upstream of that area; Saiz-Fernández et al., 2020). Red, a similar response; yellow, a similar response only in one experiment; blue, a contrasting response or proteins found exclusively in hybrid poplar *Phytophthora* response proteome. Visualized using DiVenn (Sun et al., 2019). For details, see **Supplementary Tables S7**.

The identified differentially abundant proteins were compared with two recently published datasets describing *P. cinnamomi-*responsive proteins in the infected roots of *Quercus* sp. and infected stem of *Castanea sativa* (Saiz-Fernández et al., 2022; Saiz-Fernández et al., 2020). The comparison was based on annotated Arabidopsis orthologs, identified enzymes, and protein families. The corresponding ortholog or a matching protein family was not found for 120 differentially abundant proteins, indicating that these all could be contributing to the observed tolerance (**Table S8**). For 396 hybrid poplar *Phytophthora-*responsive proteins, the matching *P. cinnamomi-*responsive proteins were found in at least one of the datasets. Most of these (337) showed a similar response in hybrid poplar to that found in oak or sweet chestnut *Phytophthora* response proteomes (**Figure 10**).

## Discussion

### Significant portion of identified *Phytophthora-*responsive proteins likely represents a non-specific biotic stress response and is not directly correlated with the observed tolerance

The described experiments were designed to identify mechanisms underlying the observed tolerance against *Phytophthora* infection reported in Ďurkovič et al. (2021). Two representatives of aggressive *Phytophthora* spp. were used in this study. As illustrated in **Figures 3**, **6**, and **7**, *P. plurivora *response was more potent, and the inoculated samples were more distant from the mock-inoculated controls. However, the main objective was the identification of mechanisms that could play a role in *Phytophthora *tolerance. For that reason, wood proteome analysis employed these treatments as two completely independent biological replicates, and most of the presented results focused on shared responses. The analysis of wood microcores of *P. plurivora* and *P. cactorum* infected trees revealed several hundred putative *Phytophthora-*responsive proteins. Metabolome and lipidome analyses confirmed that changes in the protein abundances impacted the amino acid pool, carbohydrates, secondary metabolites, and lipidome (**Figures 6**, **8**, **Table S3** and **Tables S5**, **S6**). These experiments provided independent validation for the corresponding differentially abundant enzymes. However, some of these identified proteins and metabolic pathways inherently represent a non-specific biotic stress response and are unlikely to be responsible for the observed tolerance against *Phytophthora*. The question is, how to identify proteins and metabolic processes that could have that role? To at least partially address that question, the identified differentially abundant proteins were compared with *P. cinnamomi-*responsive proteins in *Quercus* sp. and *Castanea sativa*. In both these models, *Phytophthora* is a causal agent of a destructive disease, and plants do not prevent the pathogen’s spread. Most of the identified orthologs (337) showed a similar response in hybrid poplar to that found in oak or sweet chestnut *Phytophthora* response proteomes (**Figure 10**). Only 59 proteins manifested an opposite accumulation pattern, including five proteins that were found in all three datasets (alcohol dehydrogenase Potra001097g09556.2, Aquaporin Potra187883g28547.2, Kunitz trypsin inhibitor Potra000875g07050.1, and two Stress-response A/B barrel domain-containing proteins HS1, Potra001955g15440.1 and Potri.010G150500.1.p1). It is tempting to speculate that the identified aquaporin could represent a peroxiporin (Černý et al., 2018), whose accumulation could represent a higher hydrogen peroxide flux that could correlate with an increase in the abundance of superoxide dismutase (Potra173456g27649.1).

### Candidate proteins underly metabolic pathways leading to systemic acquired resistance

The leaf proteome analysis showed that *Phytophthora* infection could have an impact on distal organs. Though the leaf response in the natural environment is prone to additional biotic and abiotic stressors, the identified proteins indicated an altered pattern in stress response proteins, including induction of jasmonic acid biosynthesis (**Figure 7**, **Table S4**). The untargeted metabolome analysis did not find any direct mediator of systemic acquired resistance (SAR, Zeier, 2021), but several candidate proteins mediate the biosynthesis of SAR metabolite precursors. A pathway of interest is the biosynthesis of lysine from aspartate that is mediated by enzymes 4-hydroxy-tetrahydrodipicolinate reductase (Potra003530g21914.1), diaminopimelate epimerase (Potra001065g09156.1), and 4-hydroxy-tetrahydrodipicolinate synthase (Potri.014G071100.1.p1), all of which were significantly accumulated in response to *P. plurivora*. Aspartic acid was 1.8-fold more abundant (p<0.1) in the infected trees, but the lysine content was below the detection limit of untargeted analysis (**Table S3**). Interestingly, lysine is a precursor for N-hydroxypipecolic acid, a metabolite that has a major role in SAR (Hartmann and Zeier, 2018).

Functional analysis performed using String highlighted an overall increase in photosynthesis-related proteins in the leaves of poplar plants inoculated with *P. plurivora* (**Figure 7C**). Indeed, trees infected with *P. plurivora* showed increased rates of net photosynthesis compared to those infected with *P. cactorum* during the growing season following the inoculation, despite the differences being not significant in all measurement days (Ďurkovič et al., 2021). To this regard, it has been suggested that infected tissues can act as a carbon sink, altering plant resource allocation patterns, which can be compensated by increased photosynthetic rates (Pagán and García-Arenal, 2018; Berezovska et al., 2021; D’Souza et al., 2021). In fact, several carbohydrates, including sucrose, showed an increased concentration in areas surrounding the necrotic tissues (**Figure 6**). These compounds could have been used by poplar trees as substrate for defense-related pathways of secondary metabolism, including lignin synthesis (Zhu et al., 2018).

### Identified CAZymes are likely candidates for promoted tolerance

Metabolome analysis of wood microcores demonstrated remarkable alteration in the hybrid poplar carbohydrate pool, including a statistically significant increase in the abundance of sucrose, the most abundant carbohydrate in the analyzed dataset. The comparison with differentially abundant proteins found in trees with failed resistance against *Phytophthora* indicated a putative role in tolerance for 13 CAZymes. The first notable candidate is trehalose-phosphate synthase (Potri.001G139500.1.p1, accumulated in response to infection). Trehalose 6-phosphate is an important signaling molecule in plants (Paul et al., 2018), and as a direct precursor to trehalose, it has been associated with promoted plant defense (Fernandez et al., 2010). The metabolome dataset also indicated that trehalose itself could be significantly accumulated in response to *Phytophthora*, but the corresponding matching metabolite did not meet the set ΔRI threshold (**Table S3**). Mannose-1-phosphate guanylyltransferase (Potra000464g02745.1, accumulated in response to *Phytophthora*) provides a substrate for cellulose biosynthesis (Lukowitz et al., 2001), which could promote cell wall thickening (Maleki et al., 2020). It is also an essential enzyme for ascorbate production, and ascorbate is well known to have a role in plant defense mechanisms (Pavet, 2005). Finally, two trifunctional UDP-*β*-L-rhamnose biosynthetic enzymes RHM1 (Potra002830g20077.1, Potri.001G383500.2.p1) were significantly accumulated in the samples from infected trees, and the accumulation pattern correlated (Pearson’s r>0.9) with a significant increase in the rhamnose pool (**Figure 6**, **Table S1**, **Table S3**). This enzyme also participates in the production of precursors for cell wall biosynthesis, and UDP-rhamnose is a vital supply for flavonol biosynthesis (Yonekura-Sakakibara et al., 2008). That is well-in-line with the results of functional enrichment that highlighted an increase in flavonoid biosynthesis in *Phytophthora-*responsive proteins (**Figure 4A**), and the accumulation of flavonoids was confirmed by metabolomic data (**Figures 6C, D**, **Table S3**). The role of flavonoids in enhancing resistance against *Phytophthora* is well known in other plant species (e.g., Cheng et al., 2015). This includes the induction of callus and tylose synthesis and the formation of crystalline structures, which minimizes the expansion of the pathogen by closing the vascular tissues (Treutter, 2006; Mierziak et al., 2014). Furthermore, epicatechin that showed the highest fold-change in hybrid poplar *Phytophthora* response metabolome was recently identified as a putative resistance marker against *P. megakarya* in leaves of cocoa (Kouam et al., 2022).

### Candidate proteins and processes mediating tolerance in *Phytophthora*-tolerant plants

The list of candidates (**Figure 10**, **Table S8**) contains two amidases (orthologs of auxin biosynthetic enzyme; Potri.013G024200.1.p1, Potra001053g09011.3; accumulated in response to *P. plurivora*) and a putative regulator of auxin transport proteins PIN1 (Aminopeptidase M1; accumulated in response to both pathogens). A pretreatment with auxin may attenuate *P. infestans* infection in *S. tuberosum* (Martínez Noël et al., 2001), and a recent study showed that despite the free auxin pool not being significantly affected, the abundance of auxin biosynthetic enzymes was positively correlated with resistance in *Solanum* accessions (Dufková et al., 2022). In this light, the identified three auxin metabolism/transport-related proteins could be part of the defense mechanism in hybrid poplar.

The set of candidate proteins includes a surprising number of ribosomal proteins (**Figure 10**, **Table S8**). Ribosomes are an integral part of cellular metabolism. Yet, despite numerous advances in the understanding of these supramolecular complexes, our knowledge about their regulatory role in adaptation and biotic stress response is limited. Previous experiments have found induced accumulation of specific ribosomal protein paralogs in response to both biotic and abiotic stimuli (reviewed e.g., Xiong et al., 2021). Ribosome composition is altered by reactive oxygen species (Dufková et al., 2019) and phytohormones (Černý et al., 2016), and that could correspond with the expected alteration in both ROS and phytohormonal homeostasis indicated in the dataset. However, the altered ribosome composition could also represent a mechanism for selective translation (Dalla Venezia et al., 2019). That regulation would contribute to the modulation of chromatin reflected in changes in abundances of multiple histone proteins and a decrease in abundance of RIN1 (Potri.004G199100.1.p), a component of chromatin remodeling complex.

The lipidome analysis found a decrease in glycerophospholipids and sphingolipids (**Figure 8A**). The set of proteins of interest includes glycerophosphodiester phosphodiesterase (Potra000423g02206.1; accumulated). This enzyme catalyzes the hydrolysis of phospholipids, and its accumulation in response to *Phytophthora* likely correlates with the observed depletion of glycerophospholipids. Its activity could correspond with the increased demand for fatty acid pool indicated by an accumulation of two orthologs of *α*-dioxygenase DOX1 (Potra000727g05695.1, Potri.008G106400.1.p1). Dioxygenases protect the plant against infection by generating oxylipins (Vicente et al., 2012).

Hybrid poplar Germin-like proteins are not found in the list of candidate proteins, but the predominant response to *Phytophthora* proximity was opposite to that found in *Quercus* sp. or *Castanea* sativa, and omics data integration highlighted their role (**Figure 9**). Germin was first identified as a putative marker of germination (Lane et al., 1992), but this protein family is vital for plant defense (Yuan et al., 2021). It has been shown that overexpression of germin-like proteins promotes resistance against plant pathogens, including *Sclerotinia sclerotiorum* and *Fusarium* (Zhang et al., 2018; Pei et al., 2019). Five germin-like proteins were found among *Phytophthora-*responsive proteins in hybrid poplar, and all showed a lower abundance in the proximity of actively growing necrosis (**Table S1**). However, the abundances of three of these (Potra001167g10122.1, Potra003370g21472.1, Potri.010G038200.1.p1) were significantly correlated with distance to the necrosis (**Figure 5C**). The seemingly counterintuitive pattern indicates that these proteins do have a role in the observed tolerance.

Finally, a family of polyphenol oxidases should be mentioned. In total, five isoforms were found to be accumulated in response to *Phytophthora* infection (three were found only in *P. plurivora* response). These enzymes that catalyze the oxidation of phenolic compounds into highly reactive quinones were previously found to be indirect regulators of cell death. Transgenic plants with a silenced gene for polyphenol oxidase developed spontaneous necrotic lesions probably due to the accumulation of tyrosine-derived metabolite tyramine (Araji et al., 2014).

## Conclusion

This work provided the first insight into molecular mechanisms underlying tolerance to *Phytophthora* in hybrid poplars. The integration of proteome, metabolome, and lipidome data revealed processes that are modulated in response to this pathogen, and the comparison with the previously published data on *Phytophthora* response in trees with compromised defense highlighted differences in *Phytophthora*-tolerant hybrid poplar. It remains to be seen how these results will be utilized, but it is tempting to speculate that the identified perspective targets could be used for improving *Phytophthora* resistance in trees in general. Not all of the most prominent candidates will likely be easily accessible for future testing and validation with the corresponding mutants. For some of these, including ribosomal proteins, that could be difficult given the sheer number of isoforms and the fact that these proteins are often integral for plant viability. Promising candidates seem to be germins and polyphenol oxidases. Similarly, a tempting target is auxin metabolism and transport, which could be probed and modified by chemical treatments.

## Data availability statement

The datasets presented in this study can be found in online repositories. The names of the repository/repositories and accession number(s) can be found below: https://www.ebi.ac.uk/pride/archive/, PXD035956, PXD035965.

## Author contributions

MC, MD, and JD designed the research. MD, IM, and JD prepared all physiological experiments, JD performed electron microscopy and contributed to the manuscript draft. MC, MB, and IS-F performed metabolome profiling. MC and MB performed metabolomics analyses. MC performed proteome and lipidome analyses, analyzed omics data, and wrote the manuscript. All authors reviewed and analyzed the results, contributed to the article, and approved the submitted version.

## Funding

This work was supported by the European Regional Development Fund, Project Phytophthora Research Centre Reg. No. CZ.02.1.01/0.0/0.0/15_003/0000453 and by funding from the Slovak scientific grant agency VEGA (1/0450/19 and 1/0108/23).

## Conflict of interest

The authors declare that the research was conducted in the absence of any commercial or financial relationships that could be construed as a potential conflict of interest.

## Publisher’s note

All claims expressed in this article are solely those of the authors and do not necessarily represent those of their affiliated organizations, or those of the publisher, the editors and the reviewers. Any product that may be evaluated in this article, or claim that may be made by its manufacturer, is not guaranteed or endorsed by the publisher.
