# Supplementary figures and images for: Defense mechanisms promoting tolerance to aggressive Phytophthora species in hybrid poplar
Source: Front Plant Sci. 2022 Oct 13;13:1018272. doi: 10.3389/fpls.2022.1018272 (PMC9621118; doi:10.3389/fpls.2022.1018272)

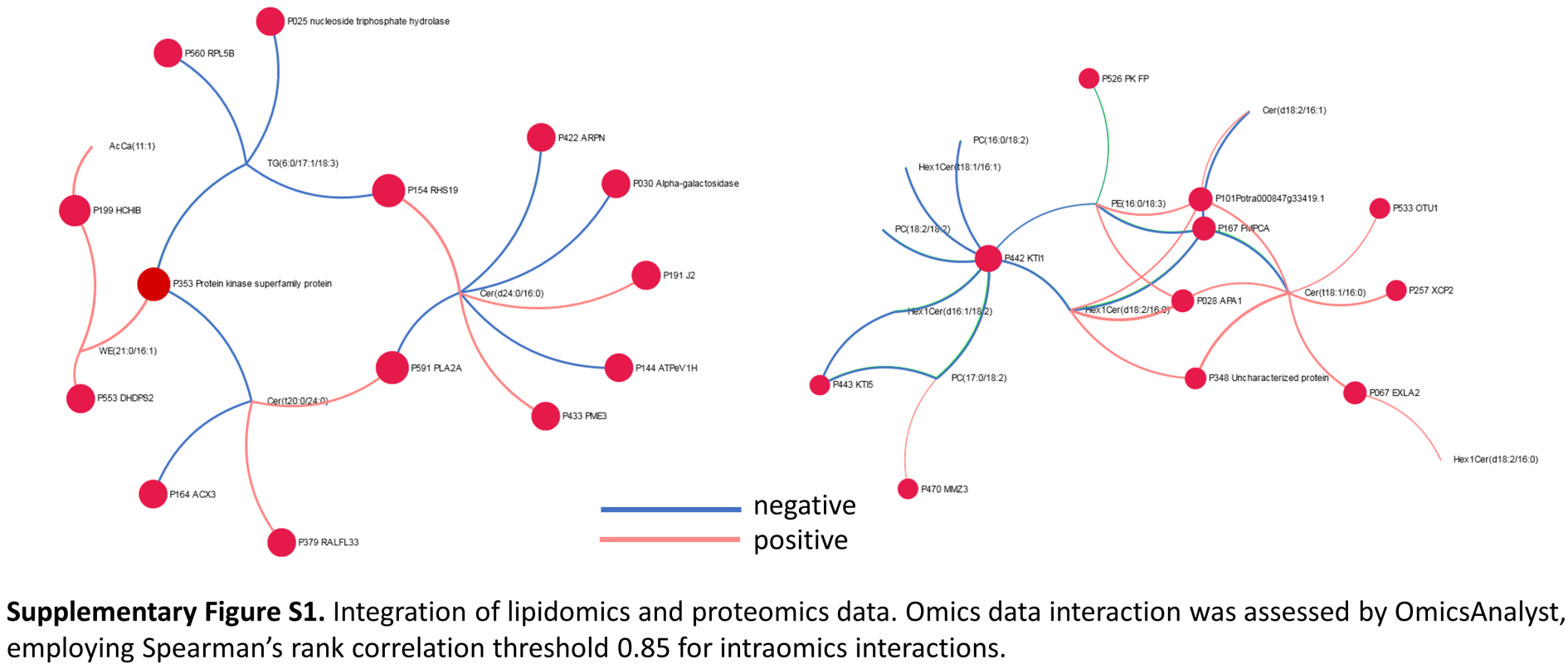

Supplement: Supplementary file 2 [file Image_1.tif]
